# Supplementary material for: Identification of a Potent Cytotoxic Pyrazole with Anti-Breast Cancer Activity That Alters Multiple Pathways
Source: Cells. 2022 Jan 12;11(2):254. doi: 10.3390/cells11020254 (PMC8773755; doi:10.3390/cells11020254)
Supplement: Supplementary file 1 [file cells-11-00254-s001.zip › cells-1468263-supplementary.pdf]

cells

MDPI

Supplementary information

Identification of a potent cytotoxic pyrazole with anti-breast cancer activity that alters multiple pathways

Denisse A. Gutierrez[1], Lisett Contreras[1], Paulina J. Villanueva[1], Edgar A. Borrego[1], Karla Morán-Santibañez[1], Jessica D. Hess[1], Rebecca DeJesus[1], Manuel Larragoity[1], Ana P. Betancourt[1], Jonathon E. Mohl[2], Elisa Robles-Escajeda[1], Khodeza Begum[1], Sourav Roy[1], Robert A. Kirken[1], Armando Varela-Ramirez[1*], Renato J. Aguilera[1*].

[1] Department OF Biological Sciences and Cellular Characterization and Biorepository Core Facility, Border Biomedical Research Center, the University of Texas at El Paso, 500 West University Avenue, El Paso, TX 79968-0519, USA.
[2] Department of Bioinformatics, the University of Texas at El Paso, 500 West University Avenue, El Paso, TX 79968-0519, USA.
* Correspondence: raguilera@utep.edu, (Tel. 915-747-6852)

Citation: Gutierrez, D.A.; Contreras, L.; Villanueva, P.J.; Borrego, E.A.; Morán-Santibañez, K.; Hess, J.D.; DeJesus, R.; Larragoity, M.; Betancourt, A.P.; Mohl, J.E.; et al. Identification of a Potent Cytotoxic Pyrazole with Anti-Breast Cancer Activity That Alters Multiple Pathways. *Cells* **2022**, *11*, 254. https://doi.org/10.3390/cells11020254

Academic Editors: Seung-Oe Lim and Alexander E. Kalyuzhny

Received: 1 November 2021
Accepted: 7 January 2022
Published: 12 January 2022

Publisher's Note: MDPI stays neutral with regard to jurisdictional claims in published maps and institutional affiliations.

Copyright: © 2022 by the authors. Submitted for possible open access publication under the terms and conditions of the Creative Commons Attribution (CC BY) license (https://creativecommons.org/licenses/by/4.0/).

Table of Contents

Supplementary Figure S1. Dose-response curve and CC_{50} value of P3C after 7 days of exposure to MDA-MB-231 cells.

Supplementary Figure S2. Representative flow cytometric dot plots were used to quantify the apoptosis and necrosis values induced by P3C on MDA-MB-231.

Supplementary Figure S3. Representative flow cytometric dot plots were required to obtain the percentages of P3C-treated MDA-MB-231 cells exhibiting caspase-3/7 activation.

Supplementary Figure S4. Representative flow cytometric dot plots were required to obtain the percentages of P3C-treated MDA-MB-231 cells exhibiting caspase-8 activation.

Supplementary Figure S5. Representative flow cytometric dot plots were necessary to obtain the percentages of P3C-treated MDA-MB-231 cells exhibiting ROS accumulation.

Supplementary Figure S6. Representative flow cytometric dot plots were used to determine the percentages of P3C-treated MDA-MB-231 cells exhibiting mitochondrial depolarization.

Supplementary Figure S7. P3C triggered PARP cleavage after 24 h of exposure to MDA-MB-231 cells.

Supplementary Figure S8. P3C compound significantly inhibits migration of MDA-MB-231 cells during the wound healing assay after 24 h of incubation.

Supplementary Figure S9. P3C-treated HeLa cells exhibited microtubule disorganization.

Supplementary Figure S10. P3C blocks cell division by disrupting microtubule and spindle fiber formation on HeLa cells undergoing mitosis.

Supplementary Figure S11. Panoramic images of HeLa cells treated with P3C and other agents.

Supplementary Figure S12. P3C provoked a reduction of Akt and p70S6k phosphorylation after 3 h of incubation with MDA-MB-231 cells.

Supplementary Table S1. Top 30 GO terms found for biological processes for the 23 up-regulated genes commonly expressed in MDA-MB-231 and MDA-MB-468 cells upon P3C exposure.

Supplementary Table S2. Canonical pathways are associated with the 28 DEGs in MDA-MB-231 and MDA-MB-468 cells.

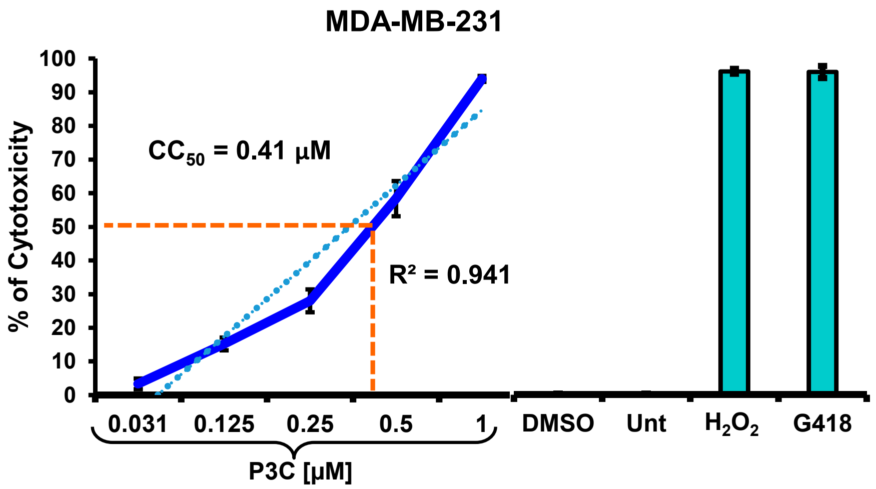

Supplementary Figure S1. Dose-response curve and CC_{50} value of P3C after 7 days of exposure to MDA-MB-231 cells. Cells were exposed to a gradient of concentrations of P3C (from 0.031 to 1 µM) for 7 days, and cytotoxicity was assessed by the differential nuclear staining assay (DNS). In this experiment, the following controls were included: 1% DMSO (vehicle), non-treated, 100 µM H_2O_2, and 10 mg/ml of G418, the last two included as positive controls for death.

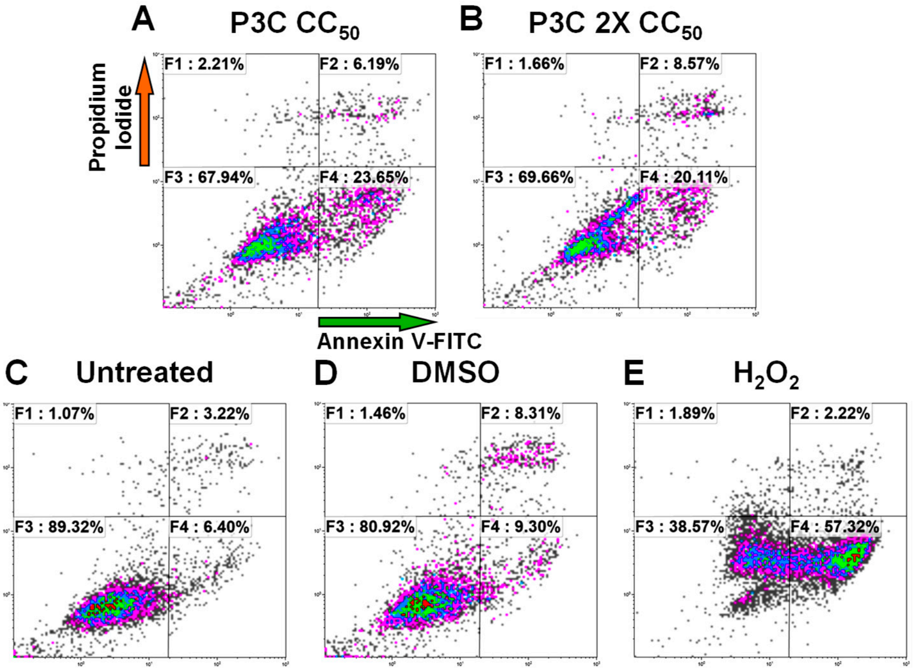

Supplementary Figure S2. Representative flow cytometric dot plots were used to quantify the apoptosis and necrosis values induced by P3C on MDA-MB-231. Cells were treated with P3C CC_{50} (A) and 2X CC_{50} (B). The following controls were included: untreated cells (C), solvent (1% DMSO) (D), and 1 mM H_2O_2 as an apoptosis inducer (E) control. Each dot in the plots represents an event (cell). The several dot colors in the plots describe only a density gradient; low-density region, grey, and high-density, red. The FL1-FITC detector is on the *x*-axis, whereas the FL2-PI detector is on the *y*-axis. Data analysis per quadrants were interpreted as follow: the left lower quadrant specifies viable cells, double-negative for Annexin V-FITC and PI; the lower right quadrant indicate Annexin V-FITC-positive, but PI-negative, early apoptotic cells; the right upper quadrant includes double-positive Annexin V-FITC and PI-positive, late apoptotic cells; the left upper quadrant designates cells just positive to PI, the necrotic cells. Around 10,000 events were acquired and analyzed using a Gallios flow cytometer and Kaluza software (Beckman Coulter).

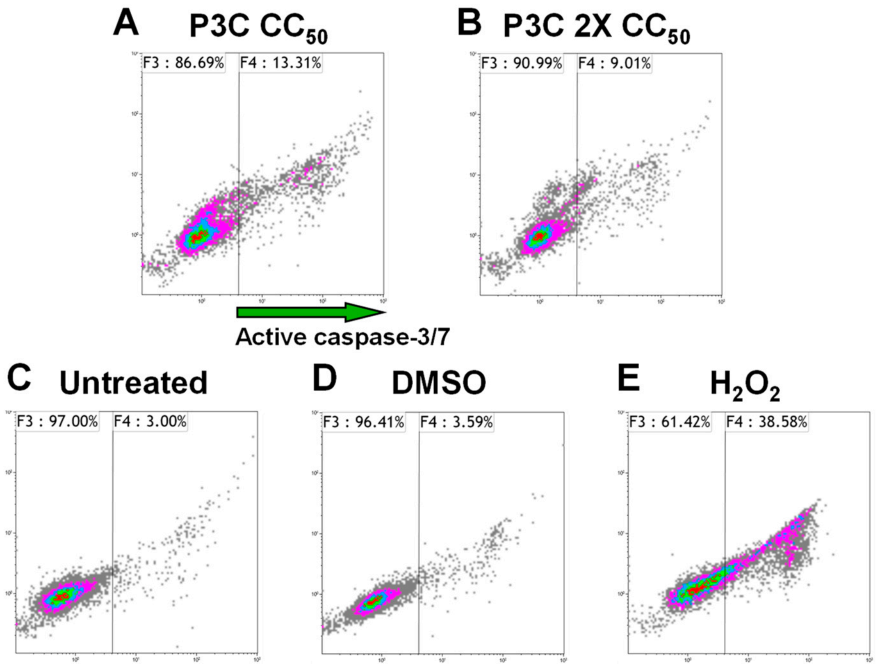

Supplementary Figure S3. Representative flow cytometric dot plots were required to obtain the percentages of P3C-treated MDA-MB-231 cells exhibiting caspase-3/7 activation. The NucView 488 caspase-3/7 fluorogenic substrate (Biotium) was used for this experiment series. Cells were treated with P3C CC_{50} (A) and 2X CC_{50} (B). The following controls were concomitantly analyzed: untreated cells (C), solvent (1% DMSO; D), and 1 mM H_2O_2 as a caspase-3/7 activator (E) control. Each dot in the plots signifies an event (cell). The plot's dot colors variety reflects only a density gradient; low-density region, grey, and high-density, red. FL1 and FL2 detectors were plotted at the *x*-axis and *y*-axis, respectively. Approximately 10,000 events were collected and analyzed per sample using Kaluza software (Beckman Coulter).

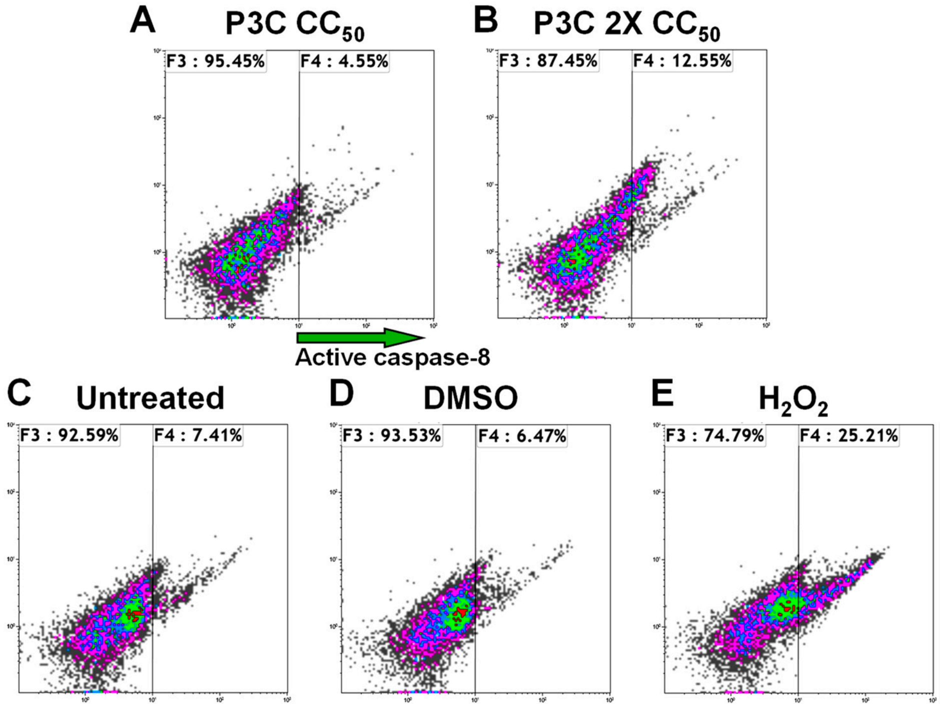

Supplementary Figure S4. Representative flow cytometric dot plots were required to obtain the percentages of P3C-treated MDA-MB-231 cells exhibiting caspase-8 activation. The IETD-AFC fluorogenic substrate (abcam) was used for this experiment series. Cells were exposed to the P3C CC_{50} (A) and 2X CC_{50} (B). The following controls were alongside analyzed: untreated cells (C), solvent (1% DMSO; D), and 1 mM H_2O_2 as a caspase-8 activator (E) control. Each dot in the plots signifies an event (cell). The plot's dot colors diversity designates only a density gradient; grey dots, low-density region, red dots, high-density. FL1 and FL2 detectors were plotted at the *x*-axis and *y*-axis, respectively. Around 10,000 events (cells) were acquired and analyzed per sample using a Gallios flow cytometer and Kaluza software (Beckman Coulter).

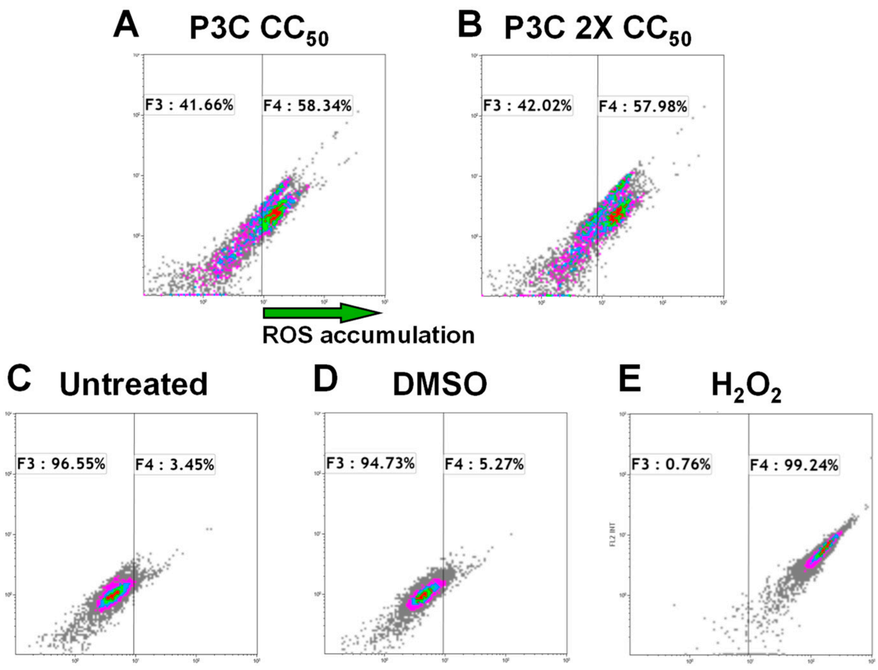

Supplementary Figure S5. Representative flow cytometric dot plots were necessary to obtain the percentages of P3C-treated MDA-MB-231 cells exhibiting ROS accumulation. For those experiments, carboxy-H2 DCFDA (6-carboxy-2′,7′-dichlorodihydrofluorescein diacetate) cell-permeant reagent (Invitrogen), and flow cytometer were used. This non-fluorescent carboxy-H2 DCFDA reagent is readily transformed to a green-fluorescent form after the acetate groups are removed from its molecule by intracellular esterases and oxidation. Cells were incubated with the P3C CC_{50} (A) and 2X CC_{50} (B). Concurrently, the following controls were analyzed: untreated cells (C), solvent (1% DMSO; D), and 1 mM H_2O_2 as an oxidative stress inducer (E) control. Each dot in the plots signifies an event (cell). The plot's dot colors variety designates only a density gradient; low-density region, grey dots, whereas high-density, red dots. FL1 and FL2 detectors were set up at the *x*- and *y*-axis. Approximately 10,000 events (cells) were collected and analyzed per sample using a Gallios flow cytometer and Kaluza analysis software (Beckman Coulter).

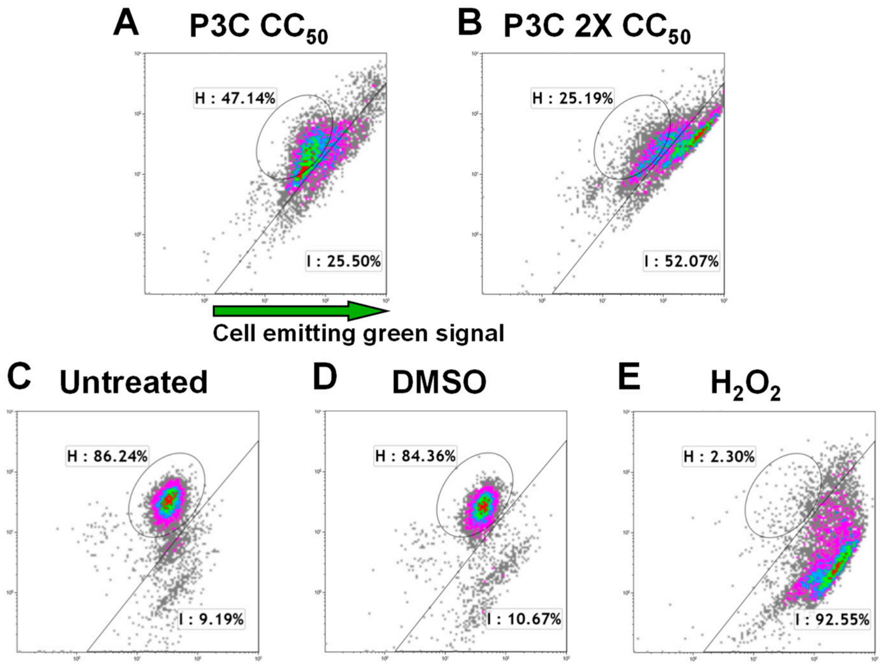

Supplementary Figure S6. Representative flow cytometric dot plots were used to determine the percentages of P3C-treated MDA-MB-231 cells exhibiting mitochondrial depolarization. The JC-1 polychromatic reagent (Invitrogen) and flow cytometer were used. When the mitochondrial membrane potential is intact, synonyms of healthy cells, the JC-1 forms aggregates, emitting red fluorescence signal (top left subpopulation). In contrast, damaged cells with depolarized mitochondria form JC-1 monomers emitting a green fluorescent signal (bottom right subpopulation). Cells were incubated with the P3C CC_{50} (A) and 2X CC_{50} (B). Simultaneously, the following controls were analyzed: untreated cells (C), solvent (1% DMSO; D), and 1mM H_2O_2 as an oxidative stress inducer (E) control. Each dot in the plots denotes an event (cell). The plot's dot colors variety designates only a density gradient; low-density region, grey dots, whereas high-density, red dots. FL1 and FL2 detectors were set up at the *x*- and *y*-axis. Around 10,000 events (cells) were collected and analyzed per sample using a Gallios flow cytometer and Kaluza analysis software (Beckman Coulter).

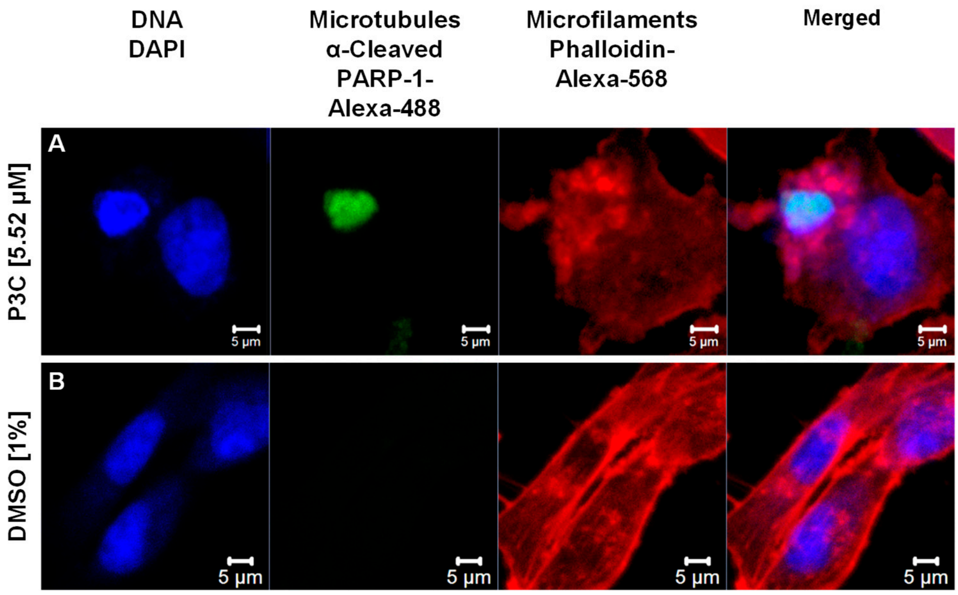

Supplementary Figure S7. P3C triggered PARP cleavage in MDA-MB-231 cells after 24 h of exposure. (A) Cells exposed to 5.52 µM of P3C (CC_{50}). (B) Cells incubated with vehicle control (1% DMSO). After treatment, cells were fixed and triple-stained with DAPI (nuclei), α-cleaved PARP-1-Alexa 488, and Phalloidin-Alexa 568 (microfilaments). The confocal immunofluorescent analysis was performed using an LSM 700 Zeiss microscope and ZEN 2009 software.

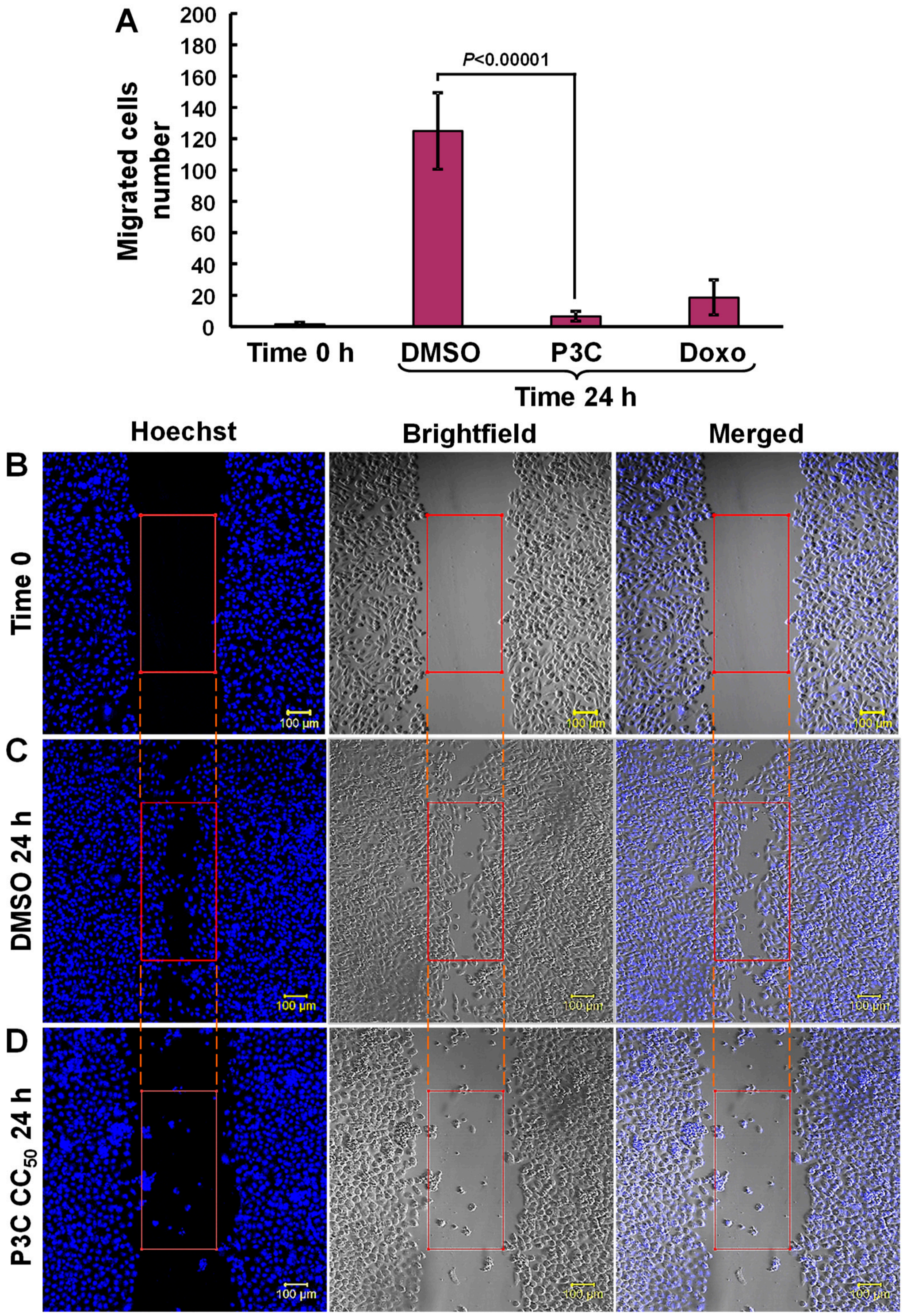

Supplementary Figure S8. P3C treatment significantly inhibits migration of MDA-MB-231 cells during the wound healing assay after 24 h of incubation. (A) Each experimental point represents the average and standard deviation of 10 independent measurements. The following controls were included: doxorubicin (Doxo), as positive of migration inhibition, and DMSO as solvent control. After 24 h, the number of cells migrating inside the defined rectangular area (demarcated by the rectangular red boundary), included in each image, was counted. Representative images showing the nuclei (blue; Hoechst), bright field illumination (phase contrast), and merged are depicted: (B) time 0 h, (C) DMSO (solvent control) 24 h, and (D) P3C experimental compound. Acquisition and analysis of the images were performed *via* Zen 2009 software (Zeiss).

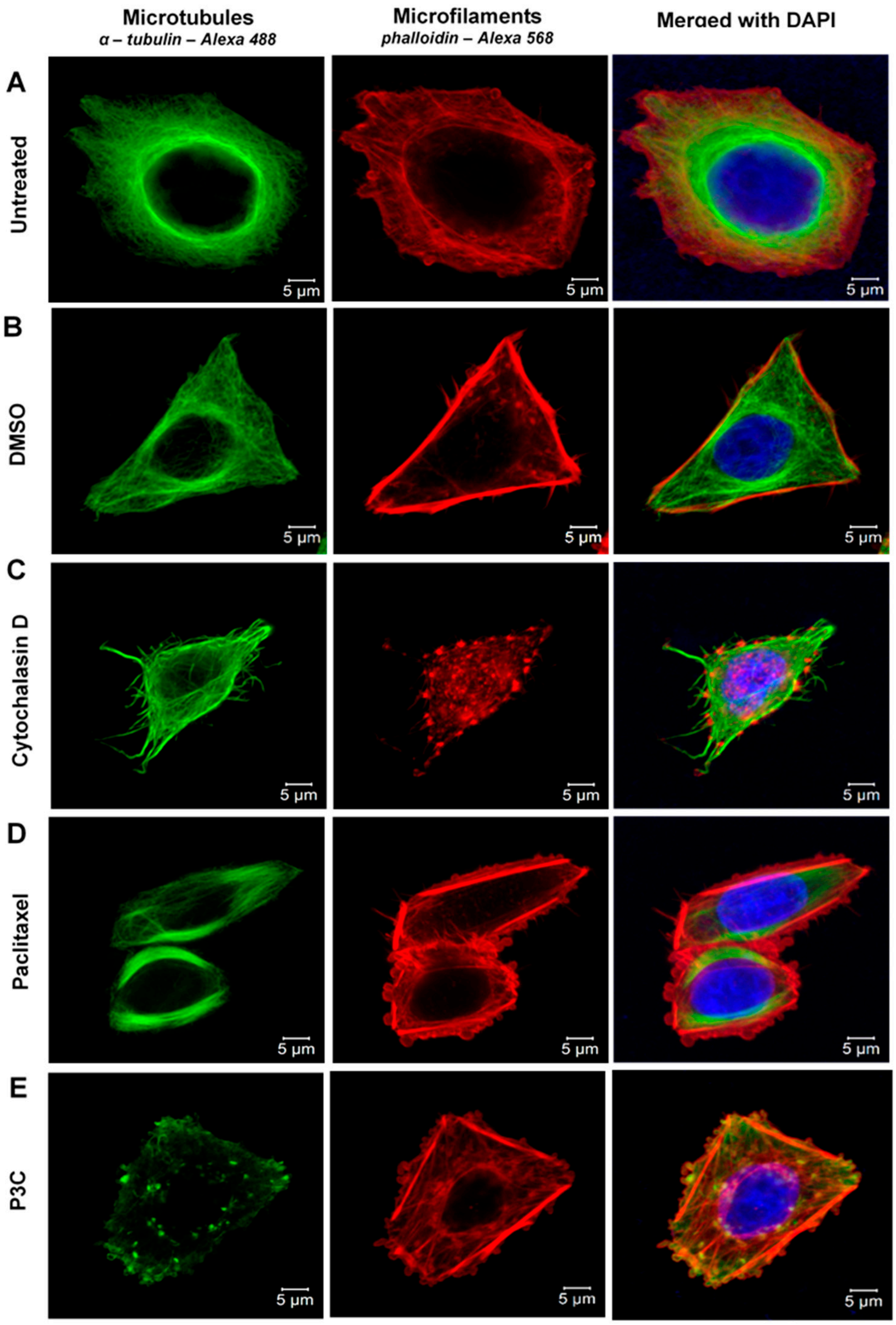

Supplementary Figure S9. Confocal microscopy images of HeLa cells demonstrated that P3C (CC_{50} 5.52 µM; panel E) perturbs microtubule organization. HeLa cells were treated and stained with α-tubulin-Alexa-488 (microtubules), Phalloidin-Alexa-568 (actin), and DAPI (nucleus). Paclitaxel (D) (spindle function inhibitor), Cytochalasin D (C) (actin polymerization inhibitor), DMSO (B) (vehicle) and untreated (A) cells were included as controls.

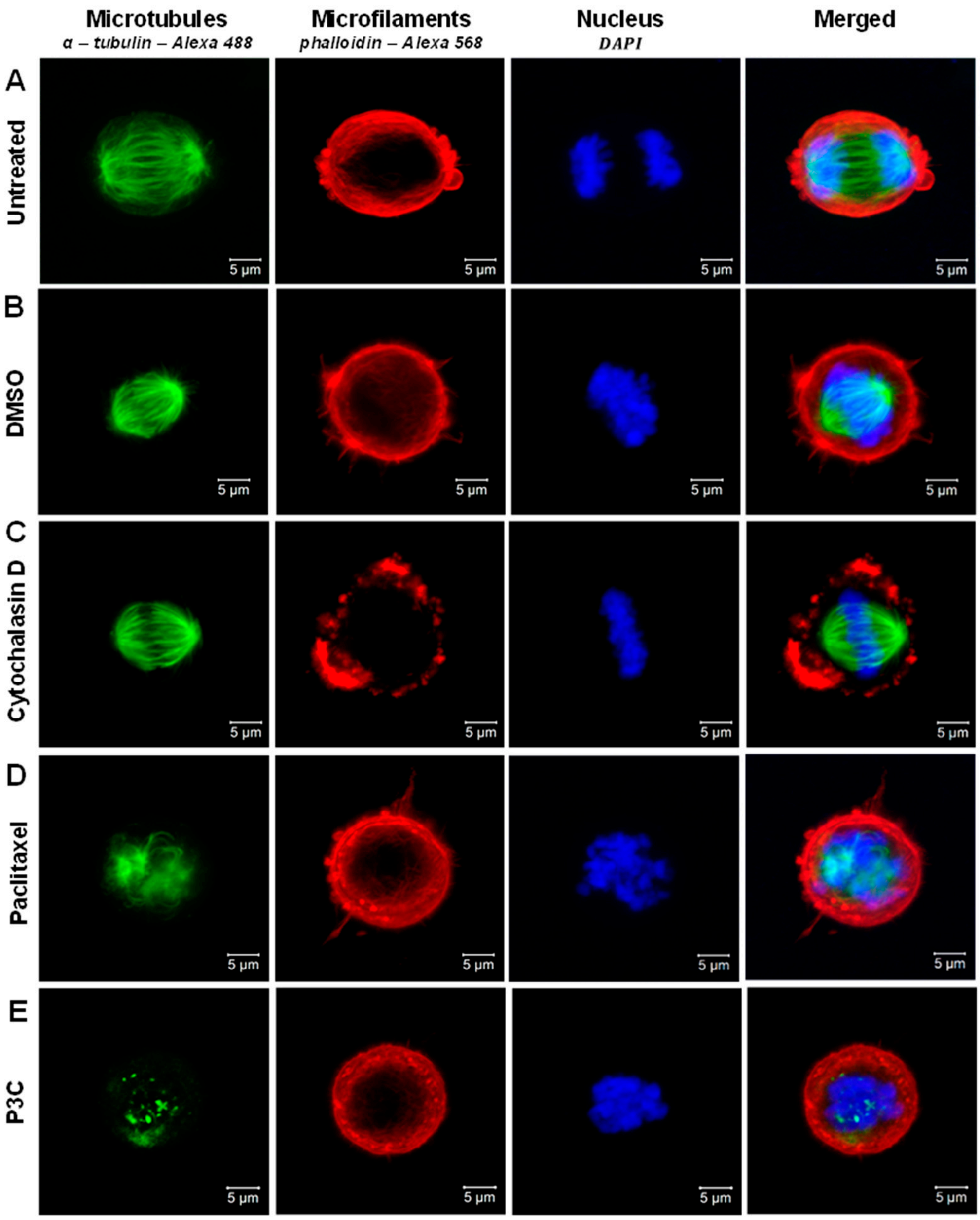

Supplementary Figure 10. **P3C blocks cell division by disrupting microtubule and spindle fiber formation on HeLa cells undergoing mitosis.** 2 h after cells were treated with P3C (CC_{50}) (E), Paclitaxel (D), Cytochalasin D (C), DMSO (B), or untreated (A) cells were immune-stained with α-tubulin-Alexa-488 (microtubules), Phalloidin-Alexa-568 (actin), and DAPI (nucleus). Cells were examined *via* confocal microscopy, and only cells undergoing cell division were imaged. Chromosome organization shown on the DAPI staining shows that cells are undergoing mitosis.

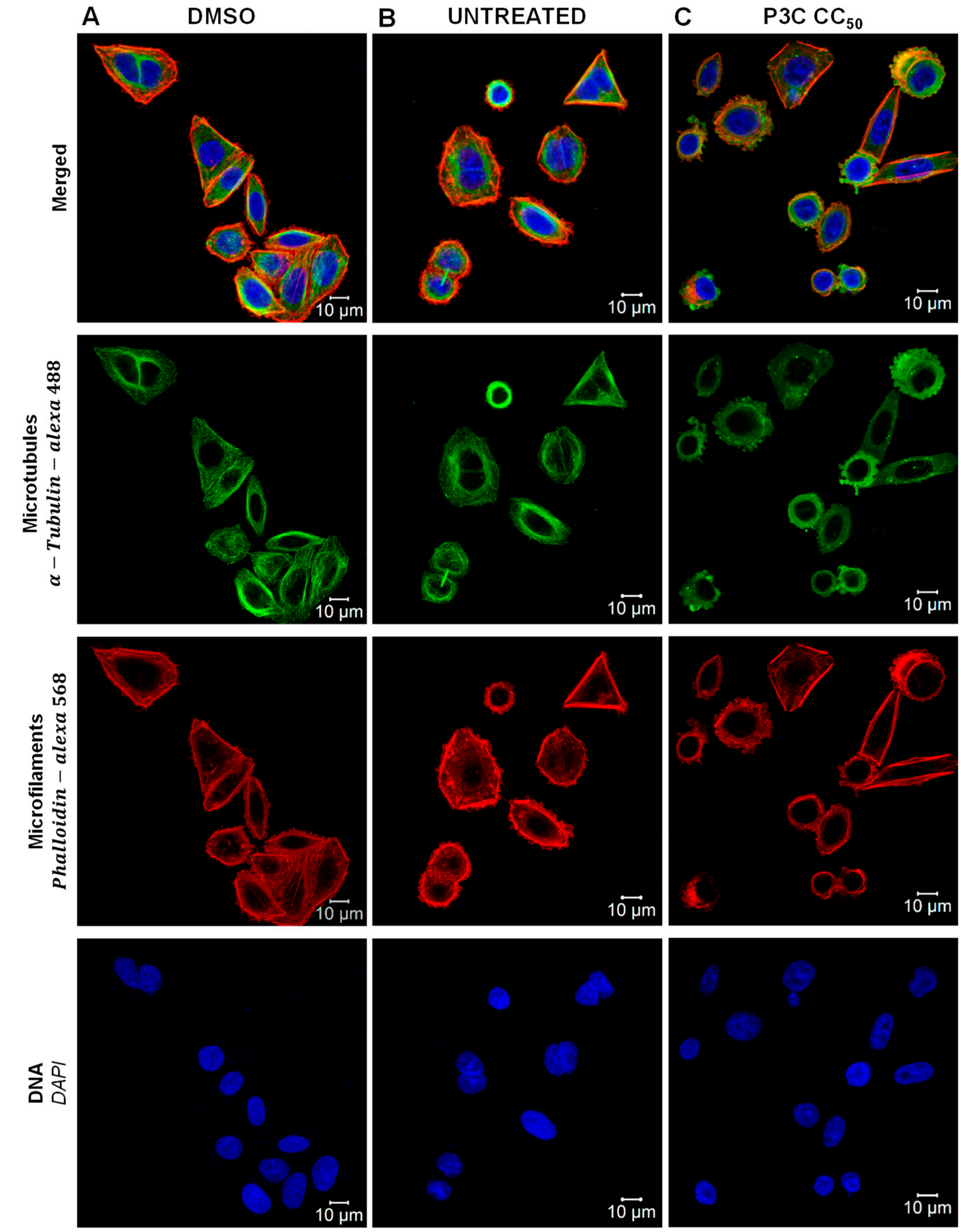

Supplementary Figure 11. **Panoramic images of HeLa cells treated with P3C (C), DMSO (A), Cytochalasin D (D), Paclitaxel (E), and untreated (B)** **(continued next page)**

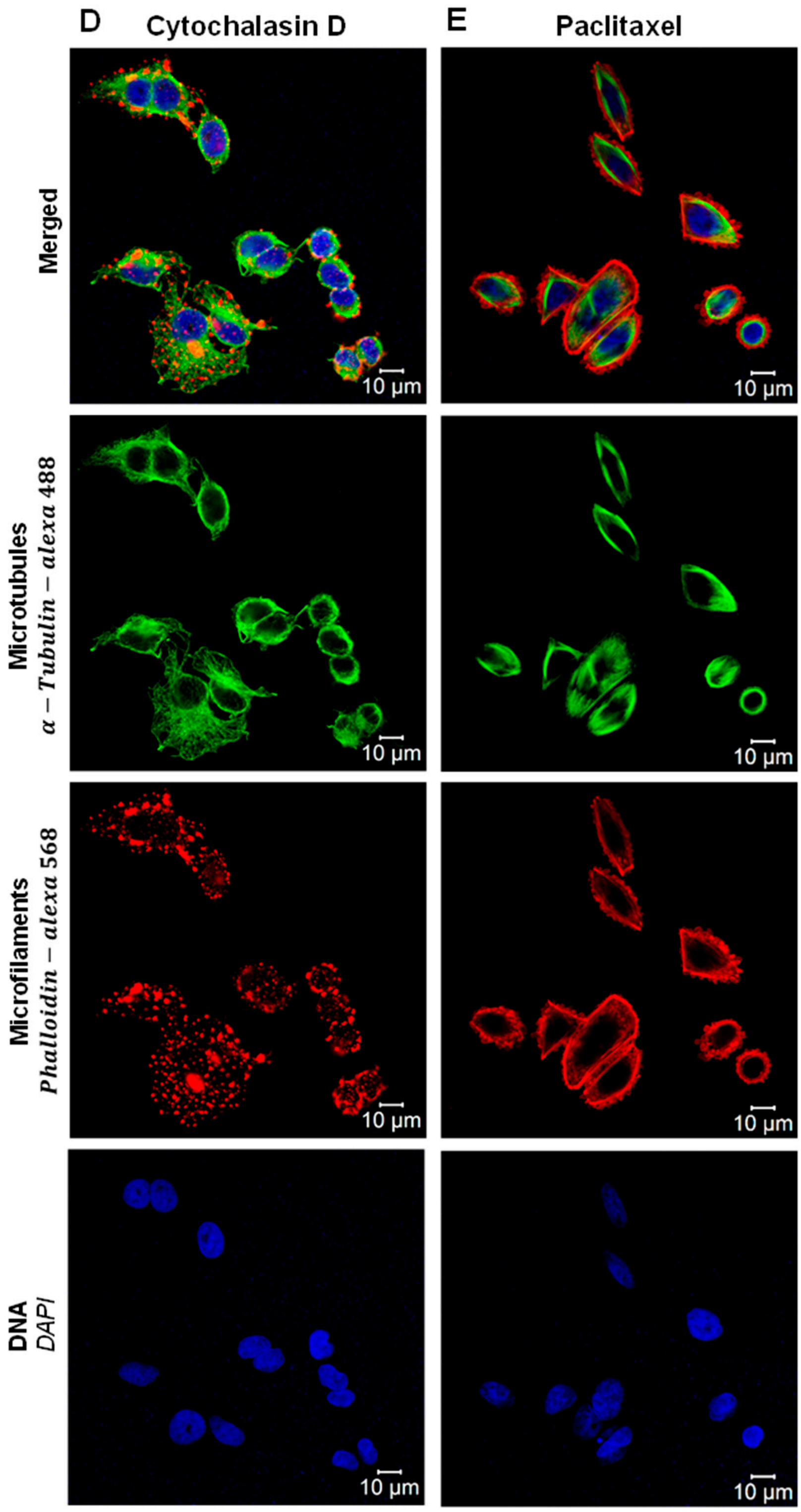

Supplementary Figure S11 (continued). **Panoramic images of HeLa cells treated with P3C (C), DMSO (A), Cytochalasin D (D), Paclitaxel (E), and untreated (B).**

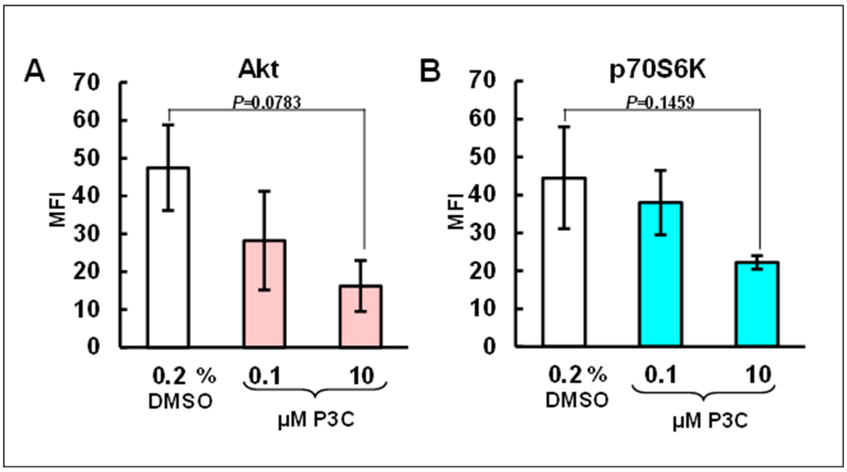

Supplementary Figure S12. P3C provoked a reduction of Akt and p70S6k phosphorylation after 3 h of incubation with MDA-MB-231 cells. Whereas P3C reduced Akt and p70S6k phosphorylation, no statistical significance was observed. A solvent control, 0.2% v/v of DMSO, was included. A bead-Based Multiplex Assay *via* the Luminex technology was employed for this series of experiments.

Supplementary Table S1. Top 30 GO terms found for biological processes for the 23 up-regulated genes commonly expressed in MDA-MB-231 and MDA-MB-468 cells upon P3C exposure*.

#term ID	Term description	Gene count	FDR	Genes involved
GO:0009605	**Response to external stimulus**	12	0.0015	HBEGF,EGR1,BTG2,REL,DDIT4,TNFRSF10D,PLAUR,DUSP10,PRDM1,GBP2,PIM1,BNIP3L
GO:0051172	Negative regulation of nitrogen compound metabolic process	12	0.0016	HBEGF,EGR1,DUSP4,MXD1,BTG2,REL,DDIT4,TNFRSF10D,PLAUR,DUSP10,PRDM1,CDKN1C
GO:0010605	negative regulation of macromolecule metabolic process	12	0.0021	HBEGF,EGR1,DUSP4,MXD1,BTG2,REL,DDIT4,TNFRSF10D,PLAUR,DUSP10,PRDM1,CDKN1C
GO:0010941	**Regulation of cell death**	10	0.0021	BIK,EGR1,BTG2,REL,DDIT4,TNFRSF10D,PLAUR,PIM1,BNIP3L,NDRG1
GO:0031324	Negative regulation of cellular metabolic process	12	0.0021	HBEGF,EGR1,DUSP4,MXD1,BTG2,REL,DDIT4,TNFRSF10D,PLAUR,DUSP10,PRDM1,CDKN1C
GO:0042127	**Regulation of cell population proliferation**	10	0.0021	HBEGF,EGR1,FOSL2,BTG2,TNFRSF10D,DUSP10,PRDM1,PIM1,NDRG1,CDKN1C
GO:0048523	Negative regulation of cellular process	15	0.0027	HBEGF,EGR1,DUSP4,MXD1,BTG2,REL,DDIT4,TNFRSF10D,PLAUR,DUSP10,PRDM1,PIM1,BNIP3L,NDRG1,CDKN1C
GO:0051248	Negative regulation of protein metabolic process	8	0.0027	HBEGF,DUSP4,BTG2,DDIT4,TNFRSF10D,PLAUR,DUSP10,CDKN1C
GO:0032268	Regulation of cellular protein metabolic process	11	0.0042	HBEGF,EGR1,DUSP4,BTG2,DDIT4,KRT17,TNFRSF10D,PLAUR,DUSP10,PIM1,CDKN1C
GO:0051707	Response to other organism	8	0.0042	EGR1,REL,DDIT4,TNFRSF10D,DUSP10,PRDM1,GBP2,BNIP3L
GO:0048856	Anatomical structure development	15	0.0063	BIK,HBEGF,EGR1,DUSP4,MXD1,FOSL2,BTG2,DDIT4,KRT17,TNFRSF10D,DUSP10,PRDM1,PLEKHO1,PIM1,NDRG1
GO:0001932	**Regulation of protein phosphorylation**	8	0.0073	HBEGF,EGR1,DUSP4,DDIT4,PLAUR,DUSP10,PIM1,CDKN1C
GO:0032269	Negative regulation of cellular protein metabolic process	7	0.0075	DUSP4,BTG2,DDIT4,TNFRSF10D,PLAUR,DUSP10,CDKN1C
GO:0050794	Regulation of cellular process	21	0.0075	BIK,HBEGF,EGR1,DUSP4,MXD1,FOSL2,TRIM36,BTG2,REL,DDIT4,KRT17,TNFRSF10D,PLAUR,DUSP10,PRDM1,PLEKHO1,GBP2,PIM1,BNIP3L,NDRG1,CDKN1C
GO:0007275	Multicellular organism development	14	0.0095	BIK,HBEGF,EGR1,DUSP4,MXD1,FOSL2,BTG2,DDIT4,KRT17,TNFRSF10D,DUSP10,PRDM1,PIM1,NDRG1
GO:0008219	**Cell death**	7	0.0095	BIK,FOSL2,DDIT4,KRT17,TNFRSF10D,PIM1,BNIP3L
GO:0031323	Regulation of cellular metabolic process	16	0.0095	HBEGF,EGR1,DUSP4,MXD1,FOSL2,BTG2,REL,DDIT4,KRT17,TNFRSF10D,PLAUR,DUSP10,PRDM1,PIM1,BNIP3L,CDKN1C
GO:0031327	Negative regulation of cellular biosynthetic process	8	0.0095	HBEGF,EGR1,MXD1,BTG2,REL,DDIT4,PRDM1,CDKN1C
GO:0035970	**Peptidyl-threonine dephosphorylation**	2	0.0095	DUSP4,DUSP10
GO:0042981	**Regulation of apoptotic process**	8	0.0095	BIK,EGR1,BTG2,TNFRSF10D,PLAUR,PIM1,BNIP3L,NDRG1
GO:0048522	Positive regulation of cellular process	14	0.0109	BIK,HBEGF,EGR1,FOSL2,BTG2,REL,DDIT4,KRT17,PLAUR,DUSP10,PRDM1,PIM1,BNIP3L,CDKN1C
GO:0001666	Response to hypoxia	4	0.0124	EGR1,DDIT4,BNIP3L,NDRG1
GO:1901214	Regulation of neuron death	4	0.0124	EGR1,BTG2,REL,DDIT4
GO:0000188	**Inactivation of MAPK activity**	2	0.0127	DUSP4,DUSP10
GO:0006950	**Response to stress**	11	0.0127	HBEGF,EGR1,BTG2,REL,DDIT4,TNFRSF10D,PLAUR,PRDM1,GBP2,BNIP3L,NDRG1
GO:0006952	Defense response	7	0.0127	EGR1,REL,DDIT4,TNFRSF10D,PRDM1,GBP2,BNIP3L
GO:0032501	Multicellular organismal process	16	0.0127	BIK,HBEGF,EGR1,DUSP4,MXD1,FOSL2,BTG2,DDIT4,KRT17,TNFRSF10D,PLAUR,TPM4,DUSP10,PRDM1,PIM1,NDRG1
GO:0051171	Regulation of nitrogen compound metabolic process	15	0.0127	HBEGF,EGR1,DUSP4,MXD1,FOSL2,BTG2,REL,DDIT4,KRT17,TNFRSF10D,PLAUR,DUSP10,PRDM1,PIM1,CDKN1C
GO:0061041	Regulation of wound healing	3	0.0127	HBEGF,PLAUR,DUSP10

* Only terms with significant enrichment are annotated (corrected *P*-values ≤0.05), and highlighted terms were considered relevant for this study. FDR: False Discovery Rate, refers to the significance of the enrichment. Corrected *P*-values are shown for multiple testing within each category using the Benjamini–Hochberg procedure.

Supplementary Table S2. Canonical pathways associated with the 28 DEGs in common in MDA-MB-231 and MDA-MB-468 cells.

Ingenuity Canonical Pathways	-log(p-value)	Ratio	Genes involved	p-value
Role of Tissue Factor in Cancer	3.46	0.0259	EGR1,HBEGF,PLAUR	3.47E-04
**SAPK/JNK Signaling**	2.19	0.0196	DUSP10,DUSP4	6.46E-03
GnRH Signaling	1.75	0.0116	EGR1,HBEGF	1.78E-02
Polyamine Regulation in Colon Cancer	1.57	0.0435	MXD1	2.69E-02
Coagulation System	1.39	0.0286	PLAUR	4.07E-02
**Cell Cycle Regulation by BTG Family Proteins**	1.37	0.027	BTG2	4.27E-02
Docosahexaenoic Acid (DHA) Signaling	1.36	0.0263	BIK	4.37E-02
Triacylglycerol Biosynthesis	1.29	0.0227	DGAT2	5.13E-02
Retinoic acid Mediated Apoptosis Signaling	1.16	0.0167	TNFRSF10D	6.92E-02
Pyridoxal 5'-phosphate Salvage Pathway	1.12	0.0152	PIM1	7.59E-02
GM-CSF Signaling	1.1	0.0143	PIM1	7.94E-02
**Protein Kinase A Signaling**	1.09	0.005	DUSP10,DUSP4	8.13E-02
Glioma Invasiveness Signaling	1.08	0.0137	PLAUR	8.32E-02
VDR/RXR Activation	1.05	0.0128	MXD1	8.91E-02
Acute Myeloid Leukemia Signaling	1	0.0112	PIM1	1.00E-01
Glucocorticoid Receptor Signaling	0.987	0.00433	CDKN1C,KRT17	1.03E-01
ErbB Signaling	0.975	0.0106	HBEGF	1.06E-01
Salvage Pathways of Pyrimidine Ribonucleotides	0.959	0.0102	PIM1	1.10E-01
Neuregulin Signaling	0.932	0.00952	HBEGF	1.17E-01
CDK5 Signaling	0.921	0.00926	EGR1	1.20E-01
Pancreatic Adenocarcinoma Signaling	0.917	0.00917	HBEGF	1.21E-01
**p38 MAPK Signaling**	0.883	0.00847	DUSP10	1.31E-01
G Beta Gamma Signaling	0.87	0.0082	HBEGF	1.35E-01
IL-12 Signaling and Production in Macrophages	0.836	0.00752	REL	1.46E-01
MSP-RON Signaling In Cancer Cells Pathway	0.833	0.00746	PLAUR	1.47E-01
**STAT3 Pathway**	0.83	0.00741	PIM1	1.48E-01
D-myo-inositol (1,4,5,6)-Tetrakisphosphate Biosynthesis	0.81	0.00704	DUSP10	1.55E-01
D-myo-inositol (3,4,5,6)-tetrakisphosphate Biosynthesis	0.81	0.00704	DUSP10	1.55E-01
3-phosphoinositide Degradation	0.772	0.00641	DUSP10	1.69E-01
D-myo-inositol-5-phosphate Metabolism	0.77	0.00637	DUSP10	1.70E-01
3-phosphoinositide Biosynthesis	0.747	0.00602	DUSP10	1.79E-01
CXCR4 Signaling	0.745	0.00599	EGR1	1.80E-01
T Cell Exhaustion Signaling Pathway	0.726	0.00571	PRDM1	1.88E-01
B Cell Receptor Signaling	0.703	0.00538	EGR1	1.98E-01
Regulation of The Epithelial-Mesenchymal Transition By Growth Factors Pathway	0.699	0.00532	EGR1	2.00E-01
Regulation of the Epithelial-Mesenchymal Transition Pathway	0.69	0.00521	EGR1	2.04E-01
RAR Activation	0.686	0.00515	REL	2.06E-01
HER-2 Signaling in Breast Cancer	0.686	0.00515	HBEGF	2.06E-01
Superpathway of Inositol Phosphate Compounds	0.676	0.00503	DUSP10	2.11E-01
IL-8 Signaling	0.674	0.005	HBEGF	2.12E-01
**ERK/MAPK Signaling**	0.67	0.00495	DUSP4	2.14E-01
Calcium Signaling	0.664	0.00485	TPM4	2.17E-01
mTOR Signaling	0.656	0.00476	DDIT4	2.21E-01
Osteoarthritis Pathway	0.638	0.00455	DDIT4	2.30E-01
cAMP-mediated signaling	0.622	0.00437	DUSP4	2.39E-01
G-Protein Coupled Receptor Signaling	0.556	0.00365	DUSP4	2.78E-01
Sirtuin Signaling Pathway	0.533	0.00344	NDRG1	2.93E-01
